# Essential Japanese Guidelines for the Prevention of Perioperative Infections in the Urological Field: 2023 Edition

**DOI:** 10.1111/iju.70026

**Published:** 2025-03-10

**Authors:** Katsumi Shigemura, Jun Kamei, Kazuyoshi Shigehara, Takuhisa Nukaya, Toshiki Etani, Yoshikazu Togo, Shingo Yamamoto

**Affiliations:** ^1^ Department of Urology Teikyo University School of Medicine Tokyo Japan; ^2^ Department of Urology Graduate School of Medicine, The University of Tokyo Tokyo Japan; ^3^ Department of Urology Kanazawa University Graduate School of Medical Science Kanazawa Japan; ^4^ Department of Urology School of Medicine, Fujita Health University Toyoake Japan; ^5^ Department of Nephro‐Urology Nagoya City University, Graduate School of Medical Sciences Nagoya Japan; ^6^ Department of Urology Kawanishi City Medical Center Kawanishi Japan; ^7^ Department of Urology Hyogo Medical University Hospital Nishinomiya Japan

## Abstract

Recently, urological surgery has undergone remarkable technological advances and these new technologies are being adapted to a wide array of medical fields. Robotic‐assisted laparoscopic procedures are not limited to radical prostatectomy, but include partial nephrectomy, pyeloplasty, radical cystectomy, and more. Perioperative infection control measures are not limited to the selection, method, and duration of antimicrobials, but include new evidence about smoking cessation, abstinence from alcohol, blood glucose control, and nutritional status assessment as well as time off just before operations. The new 2023 JUA guidelines for the prevention of perioperative infections in the urological field include general remarks and itemized discussion. We chose three standard treatments related to background questions and nine clinical questions. Although we have organized information to the best of our ability, there are still many areas where evidence is lacking. We anticipate that urology specialists will continue to report new findings and accumulate more evidence regarding unresolved areas for the next revision.

## 1 Introduction

This guideline is a short version and aims to update the 2016 version [1] and especially to focus on not only prophylactic antimicrobial agents for urological procedures and surgeries but also pre‐ and post‐surgical management. For instance, we have newly added categories such as the necessity of post‐surgical drains and the management of pre‐operative urinary tract infections (UTIs) in general remarks.

Regarding the composition, this guideline consists of general remarks and an itemized discussion, and has three background questions (BQs) and nine clinical questions (CQs). A BQ is a related question as an established standard treatment, and a CQ is a question returning new evidence to daily medical practice while considering the balance between benefits and harms. To note, regarding the biopsy, this guideline focuses on prostate biopsy.

## 2 General Remarks

### 2.1 Definition and Surgical Classification of Perioperative Infections

#### 2.1.1 BQ1: How Is Perioperative Infection Defined in Urology?

Perioperative infections include surgical site infection (SSI) and remote infection (RI).

Postoperative UTI/genital tract infection (GTI) is often difficult to clearly classify as either SSI or RI and should be treated independently as a UTI/GTI.

*Explanation*: Perioperative infections are generally defined as infections that develop within 1 month of an invasive procedure such as surgery or examinations. Perioperative infections are broadly classified into SSI, which occur at sites of surgery or procedures, and RI, which occur at sites not directly related to the surgical site. The definition of SSIs widely used today is based on the guidelines published by the Centers for Disease Control and Prevention (CDC) in 1999 [2]. The definition includes superficial incisional SSIs, deep incisional SSIs, and organ/space SSIs. According to the 1999 CDC guidelines, SSIs occur within 30 days of surgery for non‐ medical device‐indwelling procedures and within 1 year of surgery for medical device‐indwelling procedures. The diagnosis of SSI is based on clinical and laboratory findings, including purulent drainage, identification of microorganisms from cultures, physical examination findings such as pain, tenderness, local swelling, redness, heat, and fever, and the presence of an abscess on histopathologic or radiologic examination. Although the CDC definition of SSI was slightly revised in 2017 [3], the definition in Japan, including the Japan Nosocomial Infection Surveillance [4], still conforms to the previous definition of SSI in recent years. On the other hand, RI includes pneumonia, central venous catheter‐related infection, urinary catheter‐related infection, drain‐related infection, sepsis, and so on.

However, in the field of urology, where many surgical operations and procedures manipulate the urinary tract directly, there is no consensus on how to classify postoperative UTI/GTI. For example, there may be no major objection to classifying UTIs that occur after transurethral endoscopic procedures such as transurethral resection of prostate (TURP), transurethral resection of bladder tumor (TURBT), and transurethral ureterolithotripsy (TUL) as SSIs. However, it is questionable whether all UTIs/GTIs such as prostatitis, epididymitis, and pyelonephritis can be classified as SSIs in these surgeries. Considering the unique nature of many open/laparoscopic urologic surgeries, including transurethral endoscopic surgeries, it is difficult to clearly classify postoperative UTIs as either SSIs or RIs. It is also difficult to reach a consensus on whether UTIs occurring when ureteral catheters are removed long after urinary diversion, pyeloplasty, or other reconstructive procedures of the urinary tract should be counted as perioperative infections. Therefore, in the field of urology, postoperative UTIs such as pyelonephritis and cystitis, and male GTIs such as prostatitis and epididymitis, should be reported as UTI/GTI rather than classified as SSI or RI.

### 2.2 Prophylactic‐Antimicrobial Agents

#### 2.2.1 BQ2: When Is the Appropriate Time to Administer Prophylactic Antimicrobials?

Initial antimicrobials should be administered from 30 min before to just before the start of surgery (EL; IVa, RG; B).

Intraoperatively, additional doses are recommended to maintain adequate antimicrobial blood levels (EL; IVa, RG; B).

*Explanation*: The CDC guidelines published in 1999 specify that initial antimicrobials should be administered so that blood and tissue antimicrobial concentrations at the site of the surgical procedure are sufficient to prevent the growth of contaminating bacteria at the time of skin incision [2]. Although there is no high‐quality evidence that statistically demonstrates the optimal timing to administer antibiotics before the surgery, it is clear that the blood concentration of antimicrobial agents decreases with time after administration, and this guideline recommends antimicrobial administration from 30 min before the start of surgery to just before the start [2, 5]. However, fluoroquinolones and vancomycin require intravenous infusion over 60 min or more, so when these are used as prophylactic antimicrobial agents, administration should begin within 2 h before surgery [6, 7]. These agents have a relatively long half‐life, and even a slight delay between administration and the start of surgery may have little effect on intraoperative blood concentrations.

The CDC guidelines suggest that adequate blood and tissue levels of prophylactic antimicrobial agents from the time of surgery to several hours after the procedure is completed are useful for SSI prophylaxis [2]. When using β‐lactams such as penicillins and cephalosporins as prophylactic agents against SSI, it is recommended that concentrations should be maintained above the expected minimal inhibitory concentration (MIC) of the contaminating organism. Considering that the half‐life of most β‐lactams used as prophylactic agents for SSI is 1 to 2 h in blood, the antimicrobial agent should be readministered every 3 to 4 h after the start of surgery. A single dose for perioperative infection prophylaxis should include an additional intraoperative dose during prolonged surgery. On the other hand, for aminoglycoside and quinolone antimicrobial agents, since the maximum blood concentration is important (Cmax/MIC), high‐dose administration is required to achieve a higher maximum blood concentration [8] rather than dividing into low‐dose administrations. In addition, though intraoperative blood and tissue concentrations of prophylactic antimicrobial agents are affected by the amount of intraoperative blood loss and body weight, no evidence has been established regarding whether the timing of re‐dosing should be adjusted during massive blood loss or in highly obese patients.

#### 2.2.2 CQ1: If Preoperative Urine Culture Is Positive, Is Preoperative Treatment of UTI Recommended?

If UTI is present, antimicrobial agents sensitive to the causative organisms should be administered preoperatively (EL; IVa, RG; B).

If there is no disadvantage in postponing the surgery, consider postponing the surgery until the treatment of the infection is completed.

*Explanation*: Urologic surgery is characterized by the high likelihood of entering the urogenital tract or by the direct insertion of an endoscope into the urinary tract. Preoperative UTIs may increase the incidence of postoperative SSIs and UTIs due to urinary bacterial contamination of the surgical site.

It has been reported that bacteriuria at the time of surgery increases the frequency of postoperative infection in transurethral surgeries such as TURBT and TURP, and in clean‐contaminated surgeries involving the urinary tract such as radical prostatectomy [9, 10, 11]. In addition, in cases with bacteriuria at the time of surgery, the same types of bacteria causing bacteriuria are likely to be the causative organisms of SSI [12, 13]. In percutaneous nephrolithotomy for renal stones, there are reports associating preoperative UTI with the incidence of postoperative systemic inflammatory response syndrome (SIRS), and that preoperative antimicrobial therapy for 7 or more days for preoperative bacteriuria minimizes infectious complications [14, 15]. The European Association of Urology (EAU) guidelines strongly recommend preoperative urine culture, and the American Urological Association (AUA) guidelines also recommend evaluation for lower urinary tract symptoms suggesting UTI, urinalysis, and urine culture before any procedure that manipulates the urinary tract [16, 17]. These guidelines also recommend preoperative evaluation for the presence of UTI by performing urinalysis and urine culture, as well as checking for findings suggesting UTI. If UTI is confirmed preoperatively, the patient should be treated appropriately based on the culture and antimicrobial susceptibility results before surgery. However, there is little evidence on the duration of antimicrobial therapy required preoperatively to perform the surgery safely, or whether the duration needs to be modified depending on patient background and surgical procedure. If there is little disadvantage in postponing surgery, then the surgery should be considered to be postponed until the UTI treatment is completed and symptoms have resolved. In contrast, the postponement of surgery should be considered based on the conditions of each case and procedure, because it may lead to patient disadvantage or additional risk.

When surgery has to be performed with complications of UTI, the use of antimicrobial agents should not be covered by the recommendation in this guideline because such urologic procedures are treated as dirty‐infected surgery. The antimicrobial susceptibility of the bacteria causing UTI should be confirmed, and antimicrobial agents according to the results should be administered preoperatively. If UTI treatment is not considered complete by the time of surgery, antimicrobial agents targeting the causative isolate should be continued intraoperatively and postoperatively. If the UTI and bacteriuria are appropriately treated preoperatively, the usual prophylactic antimicrobial regimen described in this guideline should be followed. On the other hand, there is a lack of high‐quality evidence regarding the duration of antimicrobial administration in cases with asymptomatic bacteriuria, but there is also little evidence that prolonged postoperative antimicrobial administration is useful for the prevention of perioperative infection for these cases [18].

#### 2.2.3 CQ2: Is Postoperative Drain Placement Recommended?

Robot‐assisted radical prostatectomy (RARP) (EL; II, RG; C2), robot‐assisted partial nephrectomy (RAPN) (EL; I, RG; D), and nephrectomy/nephroureterectomy (EL; IVa, RG; C2) are not recommended for postoperative drain placement because of their lack of usefulness as SSI prophylaxis.

For radical cystectomy, the evidence is poor and inconclusive (EL; IVa, RG; C1).

The need should be considered in each case, taking into account the risk of postoperative complications.

*Explanation*: Drain placement is commonly used to detect postoperative bleeding, urinary leakage due to suturing failure, and infection in the surgical cavity at an early stage, as well as to remove fluid accumulation in the surgical cavity. A postoperative drain for SSI prophylaxis can be omitted in some gastrointestinal and gynecological surgeries [19, 20]. The CDC guidelines recommend that the drain be inserted away from the surgical incision and that it be a closed suction drain, and that it be removed as soon as possible after surgery.

### 2.3 Radical Prostatectomy

Currently, two randomized controlled trials (RCTs) including the patients who received RARP with or without lymph node (LN) dissection have demonstrated that drain placement did not reduce the risk of complications, the length of hospital stay, or the risk of rehospitalization [21, 22]. A meta‐analysis of 1783 RARP patients (1253 with drains and 530 without) treated with pelvic LN dissection found that the frequency of symptomatic lymphocele and complications was not significantly changed by the groups [23]. The risk of developing a postoperative lymphocele was greater with extended LN dissection; however, drain placement was not a predictor of the appearance of a symptomatic lymphocele at 2 weeks postoperatively [24]. The frequency of lymphocele after enlarged LN is much lower in RARP than in open radical prostatectomy [25, 26], so drainage is not always necessary in RARP even when combined with enlarged LN dissection. Routine pelvic drainage after RARP is of limited benefit in reducing complications, including SSI. However, the evidence is not sufficient because of a limited number of RCTs. The final decision to place a drain should take into account case‐specific risk factors and the skill of the surgeon.

### 2.4 Nephrectomy, Partial Nephrectomy, Pyeloplasty

Only one RCT on the usefulness of drain placement after open partial nephrectomy has been currently available. In this study, the frequency of complications, including SSI, in both groups was comparable, whereas drain‐related pain was significantly greater in the cases with a drain [27]. A meta‐analysis of 1086 patients that included open, laparoscopic, and RAPN showed no significant difference in overall complications or intervention rates between two groups with and without drain placement [28]. A retrospective study including 904 RAPN patients (546 with drains and 358 without) also reported that the absence of drains did not affect perioperative outcomes but rather shortened hospital stays [29]. In a retrospective study of 200 RAPN cases performed by a single surgeon, grade 3 or higher complications were more frequent in the drain‐placed cases [30].

On the other hand, there were no RCTs examining the usefulness of drain placement in nephrectomy or nephroureterectomy. One retrospective study including 272 patients undergoing laparoscopic donor nephrectomy demonstrated that there was no significant difference in the frequency of any complications, but the incidence of SSIs was slightly higher in cases with drain placement [31]. Similarly, the safety of non‐drain placement in robot‐assisted nephroureterectomy and laparoscopic or robot‐assisted pyeloplasty has been reported [32, 33, 34]. In partial nephrectomy, including RAPN, routine drain placement does not reduce complications including SSI. In nephrectomy and nephroureterectomy, there has been no evidence supporting the benefit of drain placement.

### 2.5 Radical Cystectomy

Only one RCT has investigated the efficacy of drain placement after open radical cystectomy with enlarged LN dissection and neobladder urinary diversion [35]. This study, including 58 patients (22 with a drain and 36 without) found no differences in the incidence of SSI or postoperative complications between the groups. Although this study had a small sample size, it provides preliminary evidence that drainage can be omitted even in open radical cystectomy. Since most cystectomies are now performed with robotic assistance, the usefulness of drain placement in robotic‐assisted cystectomy should be investigated.

## 3 Itemized Discussion

### 3.1 Open and Laparoscopic (Robot‐Assisted) Surgery

#### 3.1.1 BQ3: Is There a Difference in the Incidence of SSI Between Minimally Invasive Surgery (Laparoscopic or Robot‐Assisted Surgery) and Open Surgery?

In clean surgery, many reports show no difference between minimally invasive surgery and open surgery. Clean surgery is surgery that does not involve a breach of aseptic procedures and does not involve invasion of the digestive tract, reproductive organs, or the urinary tract without infection [2].

The incidence of SSI tends to be lower in minimally invasive surgery than in clean‐contaminated or contaminated surgeries.

*Explanation*: Laparoscopic surgery in the urological field has spread rapidly in recent years and is surpassing open surgery. Furthermore, since RARP was covered by insurance in 2012, laparoscopic surgery, including robot‐assisted surgery, has become increasingly popular. Here, minimally invasive surgery is defined as the combination of laparoscopic surgery and robot‐assisted surgery. Minimally invasive surgery is generally less invasive than open surgery, and the wounds are smaller. Although minimally invasive surgery is expected to result in fewer perioperative infections, there have been few randomized comparisons between open and minimally invasive surgery in the field of urology. In a prospective multicenter study of perioperative infections in urology, Togo et al. [36] reported that in clean surgery with a single dose or no dose of a first‐generation cephalosporin or β‐lactamase inhibitor (BLI)‐formulated penicillin antibiotic, and in clean‐contaminated surgery with a single dose of a first or second‐generation cephalosporin or BLI‐formulated penicillin for single‐dose nephrectomy (open/laparoscopic), SSI 3%/0%, RI 5%/2%, UTI 1%/1%; for partial nephrectomy (open/laparoscopic), SSI 0%/2%, RI 5%/3%, UTI 3%/3%; for radical prostatectomy (open/laparoscopic), SSI 4% /3%, RI 3%/2%, UTI 1%/4%, and for nephroureterectomy (open/laparoscopic), SSI 1%/−, RI 1%/−, and UTI 3%/−. The frequency of perioperative infections was not high, and there was no significant difference between the two groups. Furthermore, the incidence of SSI in clean surgery was 1.6% in urologic laparoscopic small incision (minimal wound endoscopy) surgery without prophylactic antimicrobial agents [37]. Although prophylactic antimicrobial agents were not mentioned, the incidence of SSIs in open partial nephrectomy and minimally invasive partial nephrectomy was reported to be 1.3%–1.4% and 0.2%–0.54%, respectively, with a significantly lower incidence of SSIs in minimally invasive partial nephrectomy [38, 39].

In a single‐center retrospective comparison of RARP and open prostatectomy, the incidence of SSI was 0.6% and 4.5% for first‐generation cephalosporins administered within 24 h as prophylactic antimicrobial agents, with a significantly lower incidence of SSI in RARP [40]. The incidence of SSI was reported to be 1.5% and 0.7% for open and minimally invasive surgery using propensity score matching in 11,183 cases of radical prostatectomy [41]. Finally, radical cystectomy and ileal conduit diversion which are contaminated surgeries, are not associated with a prophylactic antimicrobial agents.

A multicenter report the incidence of SSI ranged from 4.67% to 14% in open surgery, and from 2.95% to 3% for minimally invasive surgery [42, 43].

#### 3.1.2 CQ3: What Prophylactic Antimicrobial Administration Is Recommended for Clean Surgery?

A single dose of a first‐generation cephalosporin or penicillin with BLI is recommended (EL; III, RG; B).

*Explanation*: For upper abdominal clean surgery (adrenalectomy, nephrectomy, partial nephrectomy) or surgery in the inguinal or scrotum without open urinary tract, the main bacteria targeted are gram‐positive cocci such as *Staphylococcus aureus* , and a single dose of a first‐generation cephalosporin or penicillin with BLI is recommended (Table 1).

**TABLE 1:** Selection and duration of antimicrobial agents for the prevention of infection in the perioperative period of clean surgery.

A. Name of surgery (name of test), B. Prophylactic antimicrobials, C. Duration of administration
A. Perineal surgery (penis, scrotum, varicocele, etc.), nephrectomy, adrenalectomy, partial nephrectomy, lymph node dissection, etc.
B. First‐generation cephalosporins, BLI combination penicillin system^a^
C. Single dose: Additional dosing if surgery exceeds 3 h

*Note:* Laparoscopic and robot‐assisted surgery: same as open surgery.

^a^ Excluding PIPC/TAZ.

Although partial nephrectomy, nephroureterectomy, and partial cystectomy were conventionally considered to be classified as clean‐contaminated surgery because the urinary tract is opened, the incidence of SSI is equivalent to that of clean surgery, and the same prophylaxis as clean surgery is sufficient in the absence of UTI (Table 1) [36, 44]. A prospective multicenter study of perioperative infections in urology by Wada et al. found that in clean nephrectomy (open/laparoscopic) performed with or without a single dose of first‐generation cephalosporin or penicillin with BLI, SSI was 3%/0%, RI 5%/0%, and UTI 1%/1%, respectively. For partial nephrectomy (open/laparoscopic), SSI was 0%/2%, RI 5%/3%, and UTI 3%/3%, respectively, and there was no difference between them [45].

The incidence of SSI in clean surgery was reported to be 1.6% in urologic laparoscopic small incision (minimal wound endoscopy) surgery without prophylactic antimicrobial agents [37]. In a retrospective study by George et al. focusing on laparoscopic surgery in the field of urology, the incidence of SSI was 6.8%/2.1% for laparoscopic nephrectomy and laparoscopic adrenalectomy, respectively, when prophylactic antimicrobial agents were used within 24 h [46]. In Japan, Higuchi et al. [47] reported a 0.6% incidence of SSI with a single dose of cefazolin (CEZ) in clean surgery, and Yoshida et al. [48] reported a 3.2%/0% incidence of SSI with a single dose/no dose of penicillin with BLI. Furthermore, LaMattina et al. reported a 0.5% incidence of SSI with a single dose of CEZ in urologic laparoscopic small incision (minimal wound endoscopic) transplant nephrectomy (Table 1) [49].

In clean surgery, the majority of studies were conducted with a single dose of a first‐generation cephalosporin. The majority of studies were conducted in minimally invasive surgery, and the incidence of SSI is low, so a single dose is recommended. As mentioned earlier, there is no difference in the incidence of SSIs in clean surgery between open and minimally invasive surgery, and a single dose is appropriate even in open surgery, despite the paucity of reports. In addition, there have been very few studies of minimally invasive surgery without prophylactic antimicrobial agents, and although we cannot recommend it at this time, we believe that non‐administration may become an option in the future as the evidence builds up. Finally, although inguinal and scrotal surgeries are characteristic of urology, there are few reports on the incidence of SSIs. The incidence of this type of infection was reported to be 0% [50] or 3.5% (1.6% in the scrotum and 6.5% in the inguinal region) [51] with a single dose of first‐generation cephalosporins in a number of surgical procedures. These surgeries should also be safe with a single dose of first‐generation cephalosporins.

#### 3.1.3 CQ4: What Prophylactic Antimicrobial Administration Is Recommended for Clean‐Contaminated Surgery?

First‐ and second‐generation cephalosporins and penicillin with BLI are recommended within 24 h (EL; III, RG; B).

*Explanation*: The main target organisms are Gram‐positive cocci such as *S. aureus* and Gram‐negative rods, mainly *E. coli* . As prophylactic antimicrobial agents, first‐ and second‐generation cephalosporins and penicillin with BLI are recommended to be administered within 24 h (Table 2). In Japan, Wada et al. [45] reported that when prophylactic antimicrobial agents (first‐ and second‐generation) cephalosporins and penicillin with BLI were administered within 72 h, the incidence of SSI was 1.7%/9.5% for open radical prostatectomy and laparoscopic radical prostatectomy. Togo et al. [36] reported that the incidence of SSI was 0.9%, 3.9%, and 3.1% for nephroureterectomy, radical prostatectomy, and laparoscopic radical prostatectomy, respectively, when a single dose of prophylactic antimicrobial (first generation cephalosporin or penicillin with BLI) was used. Taoka et al. [52] and Terai et al. [53] compared the incidence of SSI for single‐dose and 24‐h administration of cefotiam (second‐generation cephalosporin) as prophylactic antimicrobial agents for open radical prostatectomy, and for 24 and 96‐h administration, and found no difference: 3.6%/2.2% and 1.2%/0%, respectively.

**TABLE 2:** Selection and duration of antimicrobial agents for prevention of perioperative infection in clean‐contaminated surgical procedures.

A. Name of surgery (name of test), B. Prophylactic antimicrobials, C. Duration of administration
A. Nephroureterectomy, Radical prostatectomy, Cystectomy, Partial cystectomy, Radical cystectomy plus cutaneous ureterostomy, Ureteral reimplantation (vesicoureteral reflux), etc.
B. First‐ and second‐generation cephalosporins and penicillin with BLI^a^
C. Within 24 h

*Note:* Laparoscopic and robot‐assisted surgery: same as open surgery.

^a^ Excluding PIPC/TAZ.

Takeyama et al. [54] reported similar results using first‐ and second‐generation cephalosporins [40]. Osmonov et al. [55] compared RARP with open surgery and reported that the incidence of SSI was significantly lower in RARP when cefalexin (first‐generation cephalosporin) and cefotaxime (third‐generation cephalosporin) were administered within 24 h. The incidence of SSI was 0.6% and 2.1% for RARP, and 4.5% and 14.4% for open surgery, respectively.

The use of prophylactic antimicrobial agents for clean‐contaminated surgery with first‐, second‐, and third‐generation cephalosporins has been reported in many cases and shows no difference in the incidence of SSIs between single/24‐h dosing and longer dosing. Based on the above results, single to 24‐h dosing is probably appropriate [36, 40, 52, 53, 54, 55]. Clean‐contaminated surgery is performed as minimally invasive procedures, and 24‐h dosing of narrow‐range first‐ and second‐generation cephalosporins is strongly recommended (Table 2). Data on single doses are not enough, and the incidence of SSI is higher compared to 24‐h dosing, and pending future development of evidence, single dosing is not recommended at this time, even for minimally invasive surgery.

Finally, in clean‐contaminated surgery where the urinary tract is opened, it is also important to search for the presence of preoperative UTI and to provide antimicrobial therapy according to the results of preoperative urine culture and antimicrobial susceptibility tests to prevent postoperative infections.

#### 3.1.4 CQ5: What Prophylactic Antimicrobial Administration Is Recommended for Contaminated Surgery (Surgery Involving the Gastrointestinal Tract)?

Cefamycin antimicrobials are recommended within 72 h (EL; IVa, RG; C1).

*Explanation*: The main target bacteria are Gram‐positive cocci such as *S. aureus* , Gram‐negative rods of *Enterobacteriaceae*, and *anaerobes*. As prophylactic agents, cephamycin is a candidate among the second‐generation cephalosporins that have antibacterial activity against anaerobic bacteria. To prevent the phenomenon of bacterial shift caused by prolonged administration, we recommend the administration of cephamycin within 72 h (Table 3).

**TABLE 3:** Selection and duration of antimicrobials for prevention of contaminated surgical perioperative infection.

A. Name of surgery (name of test), B. Prophylactic antimicrobials, C. Duration of administration
A. Radical cystectomy + urinary diversion using gastrointestinal tract
B. Cephamycin antibacterial agents such as gastrointestinal bladder enlargement
C. Within 72 h

*Note:* Laparoscopic and robot‐assisted surgery: same as open surgery.

The incidence of SSI is reported to be overwhelmingly high, ranging from 4.8% to 23.3% compared to other urological category surgeries [56, 57, 58, 59, 60, 61, 62]. The incidence of SSI in open radical cystectomy was 12.2% and 5.7% for tazobactam/piperacillin (TAZ/PIPC) (BLI‐containing anti‐ *Pseudomonas aeruginosa* penicillin) administered alone at 48 h [63] and 72 h [64], respectively, and 7.5% and 5.7% for CEZ (first‐generation cephalosporin) plus MNZ [65] or a single dose of cefmetazole (CMZ) [66] was reported to be 7.5% and 12%, respectively. The incidence of SSI was reported to be 18.1% and 20.5% for 24‐h and 72‐h administration of piperacillin (PIPC) (anti‐ *Pseudomonas aeruginosa* penicillins) alone, respectively (Table 3) [67].

Furthermore, the overall SSI rates for radical cystectomy (open surgery + minimally invasive surgery) were reported to be 22.9%/18.0% for 24‐h administration of CEZ plus metronidazole (MNZ) or ampicillin (ABPC) plus ciprofloxacin (CPFX) and MNZ [68] and 6.7% for 72‐h administration of Cefuroxime axetil (CXM‐AX) (second‐generation cephalosporin plus MNZ) (Table 3) [69].

Due to the limited number of cases, the guidelines for perioperative infection prevention in general surgery are referenced. In ascites cultures at the time of gastrointestinal perforation, the more perforation occurs in the lower gastrointestinal tract, the greater the involvement of Gram‐negative rods and anaerobes [70, 71], and when the gastrointestinal tract is used for urinary diversion, coverage of anaerobes in addition to Gram‐negative rods is important. In the “Practical Guidelines for the Appropriate Use of Antimicrobial Agents for Prevention of Postoperative Infection in general surgery,” [72] open colon resection is classified as a clean procedure, and the recommended antimicrobial agents are CMZ, flomoxef (FMOX), CEZ + MNZ, single dose to 24 h, and recommended grade A‐I. Thus, when the gastrointestinal tract is used for urinary diversion surgery in urologic surgery, the above agents should be recommended. The duration of administration should be based on the exposure time to contamination, and should be determined by the procedure, such as a short period for ileal conduit surgery and a long period for ileal neobladder.

Overseas guidelines [17] recommend a single administration of the combination of CEZ + MNZ. At present, due to insufficient evidence in Japan, a single dose of CEZ + MNZ is not recommended.

The reasons for not recommending CEZ + MNZ in this guideline are the following three points.

1. The use of two antimicrobial agents for perioperative prophylaxis is complicated.
2. According to the current Japanese data, the susceptibility of CMZ to *Bacteroides* and *Prevotella*, which are problematic anaerobic bacteria, is relatively good (60% for the former and 90% for the latter) [73].
3. MNZ should be reserved as a treatment for severe intra‐abdominal and anaerobic infections, and its use as a perioperative prophylactic antimicrobial agent is likely to promote resistance to anaerobic bacteria.

However, the rampant use of CMZ alone during contaminated surgery may accelerate the emergence of CMZ‐resistant bacteria. Depending on future trends in antimicrobial resistance, prophylactic administration of FMOX and MNZ may become necessary.

#### 3.1.5 CQ6: What Prophylactic Antimicrobial Administration Is Recommended During Surgery on Infected Wounds?

Although the answer varies according to pathological conditions, broad‐spectrum antimicrobial agents are basically recommended as empirical therapy (EL; IVb, RG; C1).

*Explanation*: This operation is classified as class IV (infected operation) by the World Health Organization. In urology, nephrectomy for pyelonephritis (including emphysematous pyelonephritis) and drainage of retroperitoneal abscess/Fournier's gangrene (necrotizing fasciitis) are considered.

## 4 Nephrectomy for Pyelonephritis (Including Emphysematous Pyelonephritis)

Pyelonephrosis and emphysematous pyelonephritis are often treated conservatively in the acute phase, followed by a standby nephrectomy. Although there are few reports in this area, the incidence of SSI is reported to be 30% when surgery is performed (open or laparoscopic surgery), and the incidence of SSI is reported to increase in the presence of emphysematous lesions with emergency surgery and when enteric bacteria are detected in culture tests (urine and blood) [74]. Although the majority of bacteria that cause SSI are Gram‐negative rods such as *E. coli* , it is desirable to start empiric therapy with antimicrobial agents that also have anti‐ *P. aeruginosa* activity and to perform de‐escalation based on culture results because of the recent increase in antimicrobial‐resistant bacteria and the prevalence of this disease in diabetic patients.

## 5 Drainage of Fournier's Gangrene (Necrotizing Fasciitis)

Because of the wide variety of organisms, including enterobacteria, anaerobes, and group A streptococci, treatment should be initiated with carbapenems or penicillin with BLI in combination with clindamycin, which inhibits toxin synthesis, and vancomycin for methicillin‐resistant *Staphylococcus aureus* control. At the same time, surgical intervention should not be delayed if it is necessary.

## 6 Endoscopic Surgery

### 6.1 CQ7: Are Prophylactic Antimicrobials Recommended for TURBT?

A single dose is recommended (EL; II, RG; B).

Consider no administration for low‐risk cases (EL; I, RG; C2).

*Explanation*: A systematic review by Bootsma et al. discussing the need for prophylactic antimicrobials in urologic surgery found low to moderate evidence suggesting no need for prophylactic antimicrobials in TURBT [75].

A recent meta‐analysis by Bausch et al. on the need for prophylactic antimicrobial agents for postoperative UTI in TURBT found no benefit for routine prophylactic antimicrobial agents in TURBT [76], and did not support the use of prophylactic antimicrobials in all TURBT patients because of the undesirable risks of antimicrobial side effects and the development of multidrug‐resistant pathogens.

In a recent multicenter RCT reported by Baten et al., 459 TURBT patients, in whom preoperative bacteriuria (> 10^4^/mL) or preoperative catheter placement (urinary catheter, double J stent, nephrostomy catheter) was excluded, no difference was found in the incidence of postoperative fever (temperature ≥ 38.3°C) in 202 patients in the prophylaxis group and 257 patients in the non‐prophylaxis group (2.9% and 3.1%, respectively; *p* = 0.44), and they reported that prophylactic antimicrobial agents could be omitted in cases with negative preoperative urinary culture or in cases with no preoperative catheter placement [77].

In a retrospective analysis of risk factors for the development of postoperative infection in TURBT, Kohada et al. reported that 21 (3.1%) of 687 TURBT patients had postoperative UTI, and that a history of pelvic radiation therapy, tumor size (≥ 2 cm), age (≥ 75 years), preoperative hospitalization (≥ 5 days), preoperative asymptomatic pyuria, and preoperative bacteriuria were associated with postoperative infection [10]. Togo et al. also analyzed risk factors for postoperative infection in transurethral surgery and found that only operative time was a significant predictor on multivariate analysis [36].

In a survey of postoperative prophylaxis in Japan [78], 37.4% of the institutions complied with the 24‐h prophylaxis period recommended by the Japanese Guidelines for the Prevention of Perioperative Infection in Urology 2006 [79] and 2016 [1], while many institutions used a slightly longer prophylaxis period. In a prospective multicenter study by Togo et al. investigating the frequency of perioperative infections in urologic surgery in Japan, postoperative UTIs were reported to be as low as 35 (2.3%) among 1497 TURBT patients who received prophylactic antimicrobial agents (first‐ or second‐generation cephalosporins or broad spectrum penicillin, single dose, i.v.) [10]. This study was conducted in a large number of patients (over 1000), and the results are considered to confirm the usefulness of a single dose in TURBT.

In TURBT, a single dose of prophylactic antimicrobial agents is recommended, but no dose could be considered for low‐risk patients with small tumor sizes and short operative times (Table 4). Although there is no clear evidence regarding the recommended agents, The Japanese Guidelines for the Prevention of Perioperative Infection in Urology 2006 recommended penicillin, first‐ and second‐generation cephalosporins, and aminoglycosides, but the related survey done by Togo et al. [36] showed that most patients received first‐ or second‐generation cephems or penicillin. In light of these results, the 2015 Guidelines for the Prevention of Perioperative Infection in Urology recommended first‐ and second‐generation cephalosporins, penicillin with BLI, and aminoglycosides, and we recommend the same agents in the present guidelines.

**TABLE 4:** Prophylactic antimicrobial agents in TURBT.

A. Name of surgery, B, Prophylactic antimicrobials, C. Duration of administration
A. TURBT
B. First‐ and second‐generation cephalosporins, penicillins with BLI,^a^ aminoglycoside
C. Single dose
Consider no dose for low risk

D. Risk factors for infection in TURBT
Preoperative bacteriuria
Preoperative urethral catheter placement
Large tumor (≥ 2 cm)
Tumors with necrosis
Surgical time (≥ 60 min)

^a^ Excluding PIPC/TAZ.

### 6.2 CQ8: Are Perioperative Prophylactic Antimicrobials Recommended for Transurethral Enucleation of the Prostate (Holmium Laser Enucleation of the Prostate [HoLEP], Transurethral Enucleation With Bipolar [TUEB])?

Prophylactic antimicrobials are recommended (EL; IVb, RG; C1).

A single dose is recommended for the duration of administration (EL; II, RG; B).

*Explanation*: The AUA [17] and Japanese guidelines [79] include information on perioperative prophylaxis for transurethral enucleation of the prostate, but the evidence for transurethral enucleation of the prostate is limited. The EAU guidelines [80] do not include information on perioperative prophylaxis for transurethral enucleation of the prostate. The AUA [17] and the Japanese guidelines [1] follow the evidence for TURP, and the EAU guidelines [80] do not include information on the need for prophylactic antimicrobial agents because of the lack of evidence for transurethral enucleation of the prostate.

In a meta‐analysis by Ahyai et al. comparing the frequency of postoperative complications in transurethral surgery in benign prostatic hyperplasia, the frequency of postoperative febrile UTI was 4.1% (0%–2%) in monopolar TURP, 2.6% (0.0%–1.5%) in bipolar TURP, and 0.9% (0.0%–0.9%) in HoLEP. Although no statistical analysis was performed, HoLEP had the lowest rate [81]. On the other hand, a subsequent systematic review and meta‐analysis by Cornu et al. found no significant difference in the incidence of postoperative UTI between HoLEP and monopolar TURP [82]. Such overseas reports suggest that the frequency of infection complications from enucleation is low or comparable to that from transurethral resection. On the other hand, the reports in Japan on the incidence of postoperative infection in transurethral enucleation of the prostate indicate rates for HoLEP of 0.7%–5.8% [37, 83, 84, 85, 86] and for TUEB of 0.3%–5.5% [36, 87, 88, 89], which are lower than the past infection rates in TURP.

In a Japanese multicenter RCT, 203 patients (125 HoLEP and 78 TUEB) undergoing transurethral enucleation of the prostate were randomized 1:1 to single‐dose or 3‐day CEZ, with results of 1.0% in the single‐dose group and 2.0% in the 3‐day group, showing no significant difference between the two treatment groups (*p* = 1.00) [90]. Therefore, in low‐risk patients without preoperative pyuria or bacteriuria, we recommend a single dose of prophylactic antimicrobial for transurethral enucleation of the prostate for the duration of the procedure (Table 5).

**TABLE 5:** Prophylactic antimicrobial agents in transurethral enucleation of the prostate.

A. Name of surgery, B. Prophylactic antimicrobials, C. Duration of administration
A. HoLEP, TUEB
B. First‐ and second‐generation cephalosporins, penicillins with BLI,^a^ aminoglycoside
C. Single dose

D. Risk factors for infection in transurethral enucleation of the prostate
Preoperative pyuria and preoperative bacteriuria
Preoperative urethral catheter placement
Residual urine (≥ 100 mL)
Surgical time (≥ 60 min)

^a^ Excluding PIPC/TAZ.

In a retrospective study of 847 HoLEP patients (87% received a single dose of 1 g of CEZ), Kyono et al. found that postoperative fever (≥ 38°C) and urosepsis (urogenital sepsis) were present in 10.3% and 0.3% of the patients, respectively, in a retrospective analysis of risk factors for the development of postoperative infection. The risk factor for postoperative fever was a positive urine culture in patients with indwelling urinary catheters in a multivariate analysis [91]. In a retrospective study of the incidence of infection and risk factors for infection, postoperative fever (≥ 38°C) and urosepsis (urogenital sepsis) occurred in 10.3% and 0.7%, respectively. In a retrospective study of 209 TUEB patients by Togo et al., the frequency of postoperative febrile UTI/GTI was 2.9%, and the risk factor for the development of postoperative infection was a preoperative residual urine volume ≥ 100 mL (*p* = 0.04) (Table 5). The incidence of postoperative infection in the single‐dose group and the multiple‐dose group was 2.9%, and the risk factor for the development of postoperative infection was a preoperative residual volume ≥ 100 mL (*p* = 0.04) (Table 5).

Although there were few studies on recommended drugs, most of the regimens reported in Japan that referred to the choice of prophylactic agents used first‐ and second‐generation cephalosporins and penicillin with BLI. In a multicenter retrospective study of HoLEP in Japan by Ishikawa et al., although the type of antimicrobial agents selected differed by institution, almost the same antimicrobial agents were selected as those used during TURP, and all institutions reported that the duration of administration was the same or shorter than that during TURP [92].

### 6.3 CQ9: Is Prophylactic Antimicrobial Therapy Recommended for TUL?

Although there is little evidence for perioperative prophylactic antimicrobial therapy for TUL, a single prophylactic antimicrobial dose is recommended (EL; II, RG; C1).

Cephalosporins, penicillin with BLI, and aminoglycosides are recommended as prophylactic antimicrobial agents (EL; VIa, RG; C1).

*Explanation*: According to previous reports, the incidence of postoperative infection after TUL surgery ranges from 4% to 11.5% [36, 93, 94]. In an RCT examining the necessity of prophylactic antimicrobial therapy in TUL, the frequency of postoperative bacteriuria was reported to be significantly lower in the group that received a single dose of levofloxacin (LVFX) [95]. An RCT comparing patients treated with a single dose of CEZ to those not treated with CEZ also found a significant difference in the frequency of postoperative bacteriuria (3.5% in the treated group vs. 35% in the non‐treated group) [96]. In a meta‐analysis of patients without preoperative UTI, a single dose of antimicrobial agents at surgery significantly reduced postoperative pyuria (RR 0.65, 95% CI 0.51–0.82) and bacteriuria (RR 0.26, 95% CI 0.12–0.60, *p* = 0.001), although the difference was not statistically significant (RR 0.26, 95% CI 0.12–0.60, p = 0.001) and a trend toward a lower incidence of febrile UTI (fUTI) was found, although there was no statistically significant difference [97]. Neither of these reports found a difference in the frequency of postoperative symptomatic UTI. Because the number of patients in both reports was small (approximately 100 cases), it is difficult to judge the pros and cons of prophylactic antimicrobial therapy based on these reports alone.

In an RCT comparing no ciprofloxacin, one dose (30 min before surgery), and two doses (first dose 30 min before surgery, additional dose within 6 h after surgery), the incidence of SIRS was statistically similar in the no‐dose group compared with the two‐dose group (9.9%, 4.9% in the one‐dose group, *p* = 0.112; 4.2% in the two‐dose group, *p* = 0.062). For stones smaller than 200 mm^2^, the incidence of SIRS was low in all three groups and did not differ significantly. However, the incidence of SIRS was significantly higher in the no‐dose group for stones larger than 200 mm^2^ (18%, single dose 4.3%, *p* = 0.036; double dose 5.5%, *p* = 0.044). The study concluded that antimicrobial therapy is not strongly recommended for stones of 200 mm^2^ or less with a negative preoperative urine culture, but that single‐dose ABP is still necessary for stones of 200 mm^2^ or more [98].

Regarding postoperative administration, a retrospective study comparing a single‐dose group with a two‐day group of antimicrobial agents reported no significant difference in the incidence of postoperative febrile UTI [99]. Another retrospective study comparing a single dose of antimicrobial with an extended postoperative administration group reported no significant difference in the incidence of postoperative febrile UTI [100].

The EAU and AUA guidelines recommend trimethoprim/sulfamethoxazole (TMP/SMX) in addition to cephalosporins and aminoglycosides, all of which are single‐dose antimicrobial agents. From the standpoint of the appropriate use of antimicrobial agents, quinolones should not be selected when the above agents are available, but they are acceptable when quinolones must be selected based on urine culture results. In cases where infected stones are expected, prophylactic antimicrobial agents should be selected based on antimicrobial susceptibilities from urine culture, and preoperative administration should be considered (Table 6).

**TABLE 6:** Prophylactic antimicrobial agents in TUL.

A. Prophylactic antimicrobials, B. Duration of administration
A. Penicillins with BLI,^a^ first‐ and second‐generation cephalosporins, aminoglycoside
B. Single dose

*Note:* In cases where preoperative urine culture is positive or infected stones were expected, prophylactic antimicrobial agents were selected based on the drug sensitivity by urine culture, and the results were used as a reference. Consider preoperative administration.

^a^ Excluding PIPC/TAZ.

## Author Contributions

**Katsumi Shigemura:** conceptualization, writing – original draft, writing – review and editing. **Jun Kamei:** methodology. **Kazuyoshi Shigehara:** validation. **Takuhisa Nukaya:** writing – original draft. **Toshiki Etani:** formal analysis. **Yoshikazu Togo:** writing – original draft. **Shingo Yamamoto:** writing – review and editing, supervision.

## Conflicts of Interest

The authors declare no conflicts of interest.
